# A novel bidirectional side exiting pledgetted suture for valve replacement surgery

**DOI:** 10.3389/fsurg.2022.920239

**Published:** 2022-07-25

**Authors:** Junnan Zheng, Henry Davies, Yiming Ni

**Affiliations:** ^1^ Department of cardiovascular surgery, the First Affiliated Hospital, Zhejiang University, Hangzhou, China; ^2^ School of medicine, University of Leeds, Leeds, United Kingdom

**Keywords:** pledgetted suture, aortic valve replacement, mitral valve replacement, suture technique, interrupted mattress suture

## Abstract

The pledgetted mattress suture is one of the most widely used suture techniques for valve replacement surgery. However, the traditional pledgetted suture has several defects including intertwining of the sutures and the pledget flipping over. Here we present a novel side exiting pledgetted suture that can overcome these defects. It offers cardiac surgeons a new alternative for valve replacement surgeries.

## Introduction

The most widely employed suture techniques for valve replacement are interrupted plegetted sutures. Pledgetted sutures that secure the prosthetic valves to the annulus during valve replacements have been shown to have a better protective effect against postoperative paravalvular leak (PVL) (1).

The classic double-headed pledgetted suture (e.g., the Ethibond 2-0 polyester pledgetted suture), has its two sutures exiting from the flat surface of the pledget in the vertical plane, making the two sutures relatively close and therefore more likely to intertwine. Furthermore, the classic suture is unidirectional as both sutures exit from one surface, resulting in the possibility of it flipping over, leaving it sitting the wrong direction. Surgeons using the classic pledgetted suture therefore spend additional time unwinding the intertwined sutures and checking that each pledget has not flipped over, thus prolonging cross-clamp time and adding to surgical risk. Here we present a novel bidirectional side exiting pledgetted suture that is less likely to intertwine with adjacent sutures and functions equally well whichever way up it lies.

## Technique and advantages

The novel suture we designed is a 2-0 double-headed bidirectional pledgetted suture that has its two sutures exiting from the middle of the short edges of the pledget rather than the flat surface.

**Figure 1:**
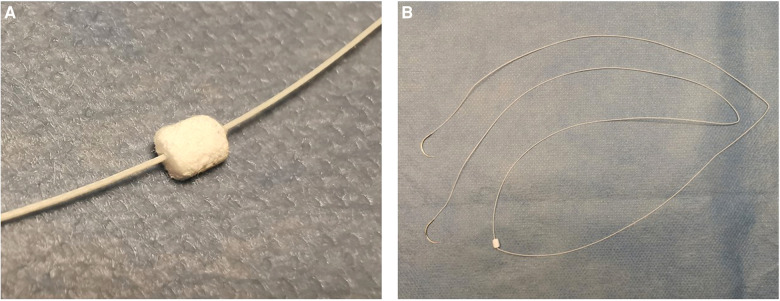
(**A**) Close-up view of the side exiting pledget. (**B**) Overall view of the novel bidirectional side exiting pledgetted suture.

The detailed design parameters of the novel side exiting pledgetted suture (Figure 1) are introduced as follows. The pledget is made of Gore-Tex materials, about 3*6*1.5 mm for mitral valve replacement (MVR), and about 3*4*1.5 mm for aortic valve replacement (AVR). The 2-0 double-headed suture is made of polyester materials, with a total length of 90 cm, with the pledget located in the middle of the suture. A 1/2 circle tapercut suture needle lies at each head of the suture (17 mm for AVR and 26 mm for MVR). The wire diameter of the polyester suture is 0.375 mm ± 0.025 mm and the diameter of the hole from which the sutures exit the pledget is 0.34 mm.

**Figure 2:**
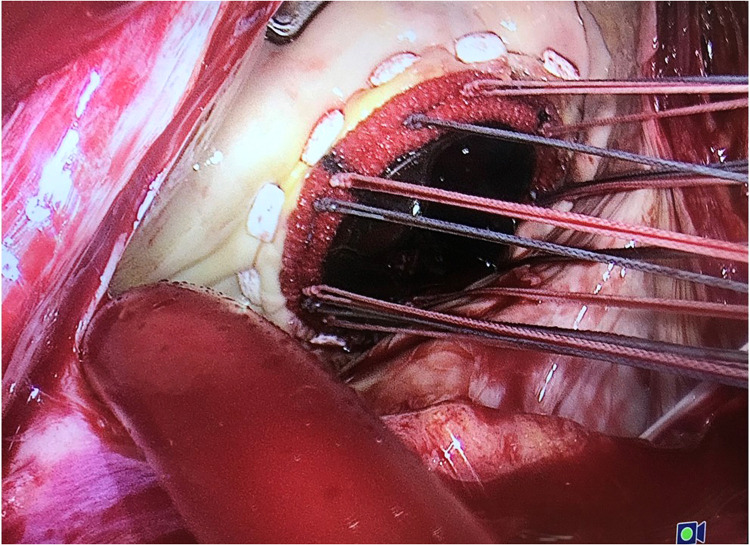
Surgical implantation of a mitral mechanical prosthesis using the novel side exiting pledgetted suture.

The novel suture we designed has three main advantages: Firstly, due to the suture side exiting nature of the pledget design, it functions equally well whichever of its flat surfaces abuts the annular surface (Figure 2), and can prevent intertwining of the adjacent sutures; secondly, the design allows the pledget adhere closely to the annular, preventing sutures from becoming hooked by the free edge of adjacent pledgets and finally, the pledget of the novel suture is less likely to interfere with the valve rotation procedure, where traditional pledgets have the tendency to catch on adjacent ones and prevent free valve rotation. This final point is especially relevant to the implantation of the SJM Master Aortic Mechanical Prosthesis, where the pivot guards of the prosthesis and the traditional pledgets combine to resist rotation similar to a rachet-pawl mechanism with the former acting as the pawls and the latter acting as rachets.

## Discussion

Surgical valve replacement remains the operation of choice to deal with most aortic and mitral valve pathology (2). The prosthesis can be inserted using continuous, semicontinuous, or interrupted sutures, with or without a pledget. Use of pledgets during valve replacement has been proven to reduce subsequent PVL (1).

The traditional pledgetted suture (e.g., the Ethibond pledgetted suture) has inherent disadvantages. First, the two sutures exit relatively close from the same surface of the pledget so readily intertwine causing the surgeon to spend extra time ensuring this has not happened and unwinding sutures that have. Also, the pledget can flip over during surgery. Especially when doing non-everting pledgetted suture, which is more often used in AVR to allow for larger prostheses. The pledget lies on the inferior surface of the annulus and thus is not visible to the operating surgeon, further adding to the risk of PVL. Moreover, the sutures of traditional pledgetted suture might become hooked by the free edge of adjacent pledgets during suturing. Therefore, surgeons must spend more time rechecking each pledget. The advantage of our suture is even more profound in minimally invasive valve replacements where view is more restricted.

There are disadvantages of our novel suture and are areas for further research. First is the possibility that the novel side sutured pledget might increase the risk of blood leaking from the suture site, as traditionally used pledgets have an inherent capability to plug blood leakage from the needle hole as the force exhibited on the pledget is more evenly distributed to a larger area immediately surrounding the hole in the tissue. Furthermore, as the distance where the sutures exit the pledget on our novel side sutured pledget is 5 mm, which is significantly larger than traditional base exiting sutured pledgets (2 mm–3 mm), there is a chance that if the two holes in the annular are too close together, our pledget could arch up and cheese wire through the annular, leading to possibly dehiscence. Therefore, the recommended needle insertion distance on the sewing ring should be equal to or slightly larger than the length of the pledget.

The novel suture was invented by our center and has been approved and put into clinical use across our nation since 2016. By the end of 2021, the novel suture has been applied in the valve replacement surgeries of more than 9,000 valves, and none had reported major adverse events due to quality issue of the suture or the pledget. From 2016 to 2021, we have applied the novel sutures in 976 mitral valve replacement and 969 aortic valve replacement surgeries in our center. Among all the prostheses, mechanical prostheses account for around 70% and the remaining 30% of patients were fitted with bioprostheses. Short-term mortality was 1.02% (20/1945). Major post-operative complications included 45 reoperations for bleeding (2.3%), 1 reoperation for valve stuck, 11 high-grade atrioventricular block requiring implantation of permanent peacemaker (0.6%), 4 severe stroke (0.2%), 10 renal failure (0.5%). During follow-up, mild-to-moderate paravalvular leak (PVL) was found in 36 patients (1.8%), mostly of the aortic position. Percutaneous transcatheter closure of the PVLs were successfully carried out in about two-thirds of the patients.

In conclusion, we described a novel bidirectional side exiting pledgetted suture that is less likely to intertwine or flip over. To date, we have used the novel sutures in multiple centres and achieved satisfactory outcomes. We hope that it can be employed by surgeons around the world, improving outcomes of patients undergoing valve replacements.

## Data Availability

The original contributions presented in the study are included in the article/Suplementary Material, further inquiries can be directed to the corresponding author/s.
